# Complement system-related genes in stomach adenocarcinoma: Prognostic signature, immune landscape, and drug resistance

**DOI:** 10.3389/fgene.2022.903421

**Published:** 2022-09-08

**Authors:** Xiaoxia Tong, Xiaohu Yang, Xiaojuan Tong, Dong Zhai, Yonglei Liu

**Affiliations:** ^1^ Experimental Research Center, Zhongshan Hospital Qingpu Branch, Fudan University, Shanghai, China; ^2^ Department of General Family Medicine, The First Affiliated Hospital of Zhejiang Chinese Medical University, Hangzhou, China; ^3^ The Third Affiliated Hospital of Zhejiang Chinese Medical University, Hangzhou, China

**Keywords:** stomach adenocarcinoma, complement system, prognostic value, gene signature, tumor immunity

## Abstract

Stomach adenocarcinoma (STAD) is one of the most common malignant tumors of the digestive tract, and its survival predictors are critical for precision medicine but have not been fully investigated. The complement system is a complex multistep cascade at the interface of innate and adaptive immunity, which augments the function of antibodies and phagocytes. This study aimed to construct and validate a CSRG signature based on TCGA (The Cancer Genome Atlas) STAD dataset and revalidated it in an external GEO (Gene Expression Omnibus) STAD cohort. Subsequently, we assessed the association of risk levels with the stromal and immune cell infiltration level in STAD using the ESTIMATE, single-sample Gene Set Enrichment Analysis (ssGSEA), and Microenvironment Cell Populations-counter (MCP-counter) algorithm. It was found that the CSRG signature, based on three genes (*SERPINE1*, *PROC*, and *CFHR3*), was significantly and independently associated with the OS in TCGA STAD patients (*p* < 0.001). Subsequently, we found that the high-risk STAD harbors more immune cell infiltration than the low-risk group, and the ESTIMATE results indicated that there exists a more stromal component in the tumor microenvironment of the high-risk groups. Compared to the low-risk group, the high-risk STAD patients had higher expressions of marker genes for immune checkpoint inhibitors (ICIs) and showed higher sensitivity to the chemotherapy agents (rapamycin, nilotinib, 5-fluorouracil, axitinib, DMOG, and JNK inhibitor VIII). The prognostic value of the CSRGs was further validated by nomogram plots, which revealed that it was superior to tumor TNM and pathologic stage. Finally, the three expression levels were evaluated in GES-1, HGC27, and AGS cells by qRT-PCR.

## Introduction

Stomach adenocarcinoma (STAD) is a major malignant tumor with an incidence rate of 5.6% and a mortality rate of 7.7% based on global cancer statistics in 2020 (1). Despite the 5-year survival rate up to 90–97% at an early stage, the 5-year survival rate is less than 30% in patients with advanced-stage STAD (2).

STAD development is complicated, involving many factors and steps, such as genetic factors, *H. pylori* infection, smoking, or environmental factors (Liu et al., 2020; Han et al., 2021). The malignant phenotypes of STAD are defined not only by the intrinsic heterogeneity of tumor cells but also by the stromal and immune cells within the tumor microenvironment (TME) (Zeng et al., 2021). Several studies on malignant solid tumors have indicated the importance of non-neoplastic stromal cells, particularly cancer-associated fibroblasts, interacting with the tumor cells that contribute to tumor formation and spread (Guo et al., 2020). Tumor-associated immune cells, primarily T cells, can produce cytokines that promote tumor angiogenesis and migration (Ren et al., 2020).

A complement system is an important innate immune system that protects the host against invading pathogens (Ueda et al., 2017; Garred et al., 2021). It has traditionally been considered as a complex network of proteins that respond rapidly to foreign pathogens and triggering inflammatory reactions (Conigliaro et al., 2019). However, activation of the complement system in the TME may promote tumor progression (Afshar-Kharghan, 2017). Recent studies have reported that complement C3 overexpression can activate the JAK2/STAT3 pathway and correlate with stomach adenocarcinoma progression (Yuan et al., 2020). Chen et al. (2018) indicated that the complement C5a/C5aR pathway can promote stomach adenocarcinoma progression by suppressing p21/p-p21 expression *via* activation of PI3K/AKT signaling. However, whether these complement system-related genes are correlated with the prognosis of STAD patients remains to be explored.

Herein, we collected the RNA-seq data and the corresponding clinical data from public databases in order to ascertain the association of complement system-related gene (CSRG) expression with STAD overall survival (OS) and treatment sensitivity and explore the possible association of immune cell infiltration with complement system gene expression.

## Methods and materials

### Cohorts and complement system-related gene selection

The CSRGs were selected according to the pathway definition in *Human Biological Pathway Unification* (https://pathcards.genecards.org/), *the HUGO Gene Nomenclature Committee* (https://www.genenames.org), and *the Molecular Signatures Database* (http://software.broadinstitute.org/gsea/msigdb/index.jsp) by the key word “complement”. After removing the duplicated genes, 248 genes remained and are provided in Supplementary Table S1. TCGA STAD cohort was downloaded from the University of California Santa Cruz Xena webserver in TCGA projects (https://xenabrowser.net/datapages/?cohort=GDC%20TCGA%20Stomach%20Cancer%20(STAD)&removeHub=https%3A%2F%2Fxena.treehouse.gi.ucsc.edu%3A443). Samples without follow-up information or with follow-up time less than 1 day were excluded. A total of 350 stomach adenocarcinoma patients in TCGA cohort were subsequently used for further analysis. In addition, another independent cohort, GSE84437, which contained 433 STAD cases, was retrieved from the Gene Expression Omnibus database for external validation (https://www.ncbi.nlm.nih.gov/geo/query/acc.cgi).

#### Construction and validation of a prognostic complement system-related gene signature

EdgeR is a package for the analysis of digital gene expression data arising from RNA sequencing technologies, such as serial analysis of gene expression (SAGE), cap analysis of gene expression (CAGE) and deep sequencing, and Tag-seq or RNA-seq, with emphasis on testing for differential expression. Thus, we used the “edgeR” R package to identify the differentially expressed genes (DEGs) between tumor tissues and adjacent normal tissues. In this study, gene sets with false discovery rates (FDRs) < 0.05 and with the threshold of |logFC|>1 were defined as DEGs.

Univariate and multivariate Cox regression analyses were conducted in TCGA cohort to screen complement system-related genes (CSRGs) significantly associated with the OS of TCGA STAD cohort. Only genes that showed significant *p* < 0.05 in the multivariate and univariate regression were considered as potential survival-related CSRGs. Then, the overlapping prognostic DEGs were selected. These genes were used to construct the risk model. Stepwise multivariate Cox regression with Akaike information criterion (AIC) was performed to obtain the risk model, and the lowest value of AIC provided the sensitivity and specificity. Subsequently, three genes (*SERPINE1*, *PROC*, and *CFHR3*) were selected. The risk score of each patient was calculated by the following formula: risk score = $\Sigma_{i=1}^{n}$ Coefi ∗Xi. Coefi represents the coefficient, and Xi indicates the expression of each significant gene. According to the risk score, all patients in TCGA cohort and GSE84437 cohort were stratified into high-risk and low-risk groups based on the optimal cut-off value of the risk score. The optimal cut-off value of the risk score was determined by the “surv_cutpoint” function of the “survminer” R package. Then, to verify the predictive power of the risk model, the “survivalROC” R package was used to conduct time-dependent ROC curve analyses.

#### Functional analysis of the differentially expressed genes

To further understand the potential function of DEGs, the Gene Ontology (GO) analysis and the Kyoto Encyclopedia of Genes and Genomes (KEGG) analysis were employed using TCGA data to understand the potential function of DEGs using DAVID online analyses.

#### Stromal and immune score determination for the stomach adenocarcinoma microenvironment

ESTIMATE is a tool for analyzing TCGA data and GEO data to estimate infiltrating stromal cells, immune cells, and tumor purity in tumor tissues based on gene expression profiles (Yoshihara et al., 2013). Herein, the ESTIMATE algorithm was applied to the normalized expression data for estimating the immune score, stromal score, and tumor purity for each STAD patient in TCGA cohort and GSE84437 cohort.

#### Immune cell enrichment analysis

The infiltrating immune cells in the tumor microenvironment were estimated by the SSGSEA and MCP-counter algorithms using TCGA data and GEO data. The MCPcounter was performed within R, as previously described (Becht et al., 2016). The SSGSEA method was performed by its package “gsva” within R, as previously described (Newman et al., 2015). Immune cell marker gene expression information of 28 subsets was obtained from the literature published by Charoentong et al. (2017). The Wilcoxon test was used to compare the differences in immune cell subtypes between the high-risk and low-risk groups.

#### Significance of the complement system-related gene-based signature in chemotherapy and immunotherapy

In order to predict the response of STAD patients in the two different risk groups to chemotherapy drugs, the “pRRophetic” R package was used to assess the half-maximal inhibitory concentration (IC50) for each STAD patient based on the Genomics of Drug Sensitivity in Cancer (GDSC) database (Geeleher et al., 2014). As several chemotherapy agents have been proven to be ineffective for advanced STAD patients, they are being replaced by immunotherapy agents, particularly the most promising ICIs, so we compared the differences in the expression levels of ICI marker genes and chemokines.

#### Enrichment analysis by gene set enrichment analysis

In order to clarify the differences of enriched pathways between the low-risk and high-risk groups, Gene Set Enrichment Analysis (GSEA) software (version 4.2.3) was utilized for TCGA data to carry out the Kyoto Encyclopedia of Genes and Genomes (KEGG) pathway enrichment analysis. The random combination was set for 1,000 times.

#### Nomogram construction and validation

After testing for collinearity, all relevant clinical characteristics and risk score were included in the construction of a prognostic nomogram to predict 1-, 3-, and 5-year overall survival of stomach adenocarcinoma patients in TCGA dataset. The concordance index (C‐index) was used to evaluate the discriminative capacity of the nomogram, and a higher C‐index suggested a superior discriminative capacity for survival outcomes. The nomogram was validated based on the internal (TCGA cohort) and external (GEO cohort) calibration measurements. The calibration curve approach to 45-degree diagonal line shows perfect predictive capability.

#### Cell culture

We obtained gastric cancer cell lines AGS and HGC27 and the normal human gastric epithelial cell line GES-1 from the Cell Bank of Chinese Academy of Sciences (Shanghai, China). All the cells were cultured in RPMI-1640 medium supplemented with 10% fetal bovine serum (Gibco, Waltham, MA) at 37°C with 5% CO_2_.

#### Quantitative real-time PCR

RNA was extracted using the TRIzol reagent (Invitrogen, United States) in accordance with the manufacturer’s protocol. Reverse transcription of RNA to cDNA was carried out according to the manufacturer’s instructions using the PrimeScript RT Reagent Kit (TaKaRa, China). Then, quantitative real-time PCR was performed with TB Green Ex Taq (Takara, China) using Applied Biosystems Prism 7500 system with customized sequences of the primers (Supplementary Table S2). The 2^−ΔΔCt^ statistic was used to calculate the expression levels of genes.

## Results

### Identification of prognostic complement system-related differentially expressed genes in The Cancer Genome Atlas cohort

**FIGURE 1:**
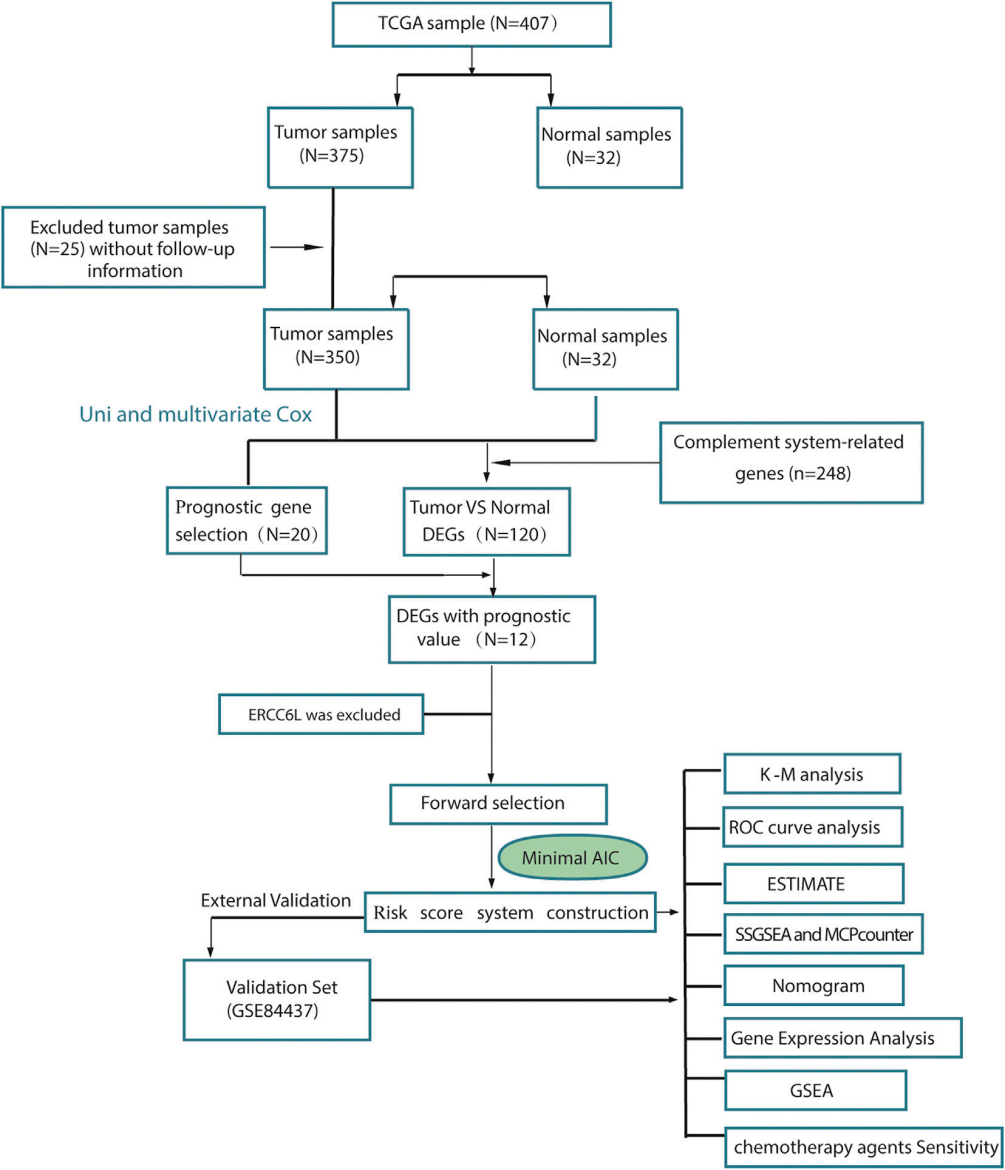
Flow diagram of data collection and analysis in the present study.

**FIGURE 2:**
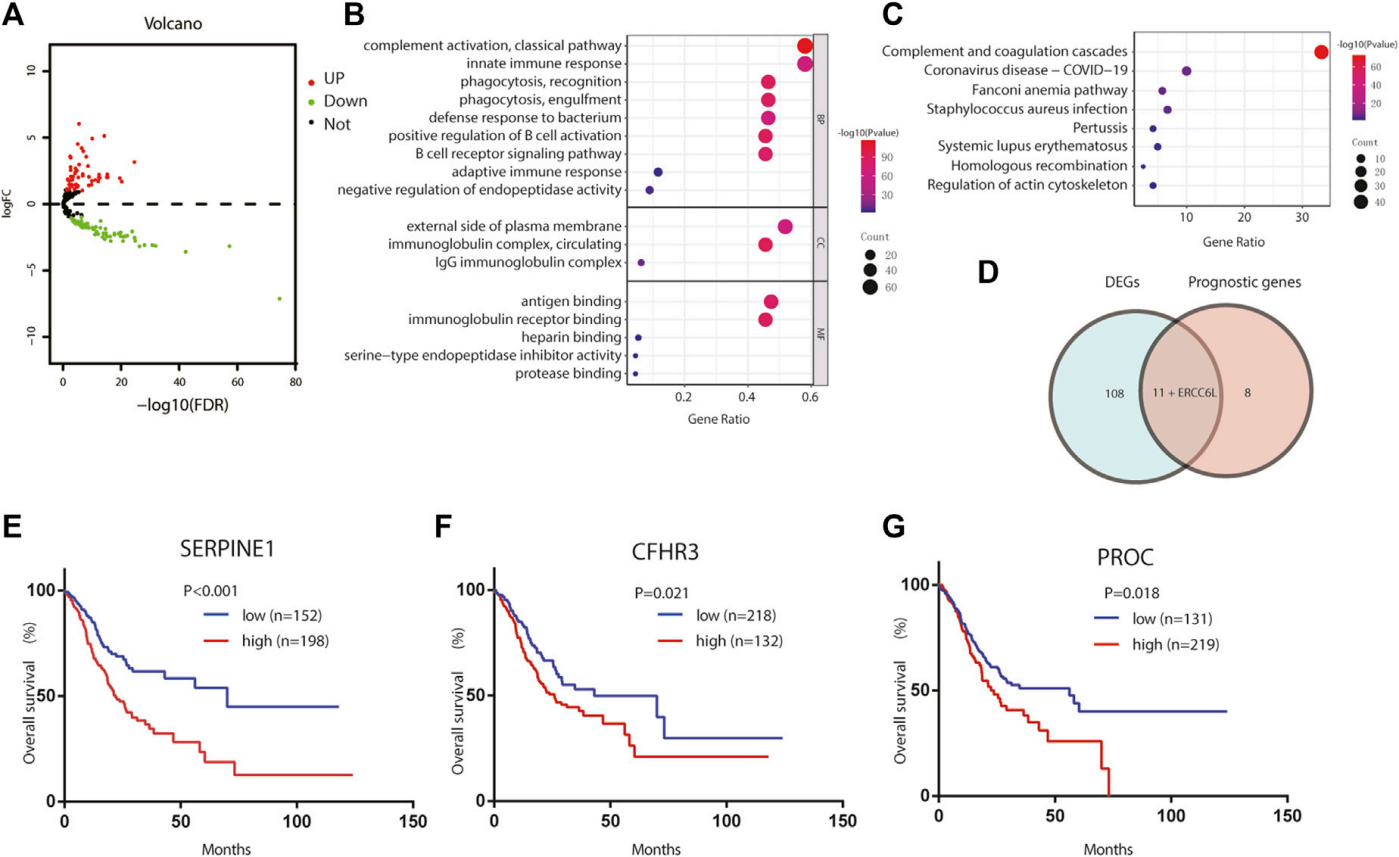
Identification of the candidate complement system-related genes in TCGA cohort. **(A)** Volcano plots visualize the complement system-related DEGs in TCGA STAD. **(B)** GO analysis of complement system-related DEGs. **(C)** KEGG analysis of complement system-related DEGs. **(D)** Venn diagram to identify DEGs between tumor and adjacent normal tissue that were correlated with OS. **(E–G)** Prognostic values of **(E)** SERPINE1, **(F)** CFHR3, and **(G)** PROC in TCGA STAD. GO, Gene Ontology; KEGG, Kyoto Encyclopedia of Genes and Genomes; DEGs, differentially expressed genes; OS, overall survival.

To describe our study more clearly, a flow chart of the analysis procedure was developed (Figure 1), and the basic characteristics of the STAD patients are shown in Table 1. In the present study, 120 DEGs in TCGA dataset were identified. Then, GO and KEGG analyses were performed to better understand the key roles of DEGs using DAVID online analyses. Of the DEGs, 12 were correlated with OS in the univariate and multivariate Cox regression analyses (Figures 2A–D). Unreasonably, ERCC6L was upregulated in tumor samples, but its expression predicted a better prognosis in the univariate and multivariate Cox regression analyses, so it was excluded from further study. A total of 11 prognostic complement system-related DEGs were preserved (all FDR < 0.05 and *p* < 0.05).

**TABLE 1:** Basic characteristics of stomach adenocarcinoma patients.

Characteristic	Group	TCGA cohort (*N* = 350)	GSE84437 cohort (*N* = 433)
		No (%)	No (%)
Age	≤ 65	161 (46.0)	283 (65.4)
	>65	189 (54.0)	150 (34.6)
Gender	Female	124 (35.4)	137 (31.6)
	Male	226 (64.6)	296 (68.4)
Pathologic stage	I	46 (13.7)	–
	II	110 (32.7)	–
	III	145 (43.2)	–
	IV	35 (10.4)	–
T	T1	16 (4.6)	11 (2.5)
	T2	74 (21.4)	38 (8.8)
	T3	161 (46.5)	92 (21.2)
	T4	95 (27.5)	292 (67.4)
N	N0	105 (30.8)	–
	N1	93 (27.3)	–
	N2	72 (21.1)	–
	N3	71 (20.8)	–
M	M0	312 (93.1)	–
	M1	23 (6.9)	–
Survival status	Dead	146 (41.7)	209 (48.3)
	Alive	204 (58.3)	224 (51.7)

### Construction of the risk score system

**FIGURE 3:**
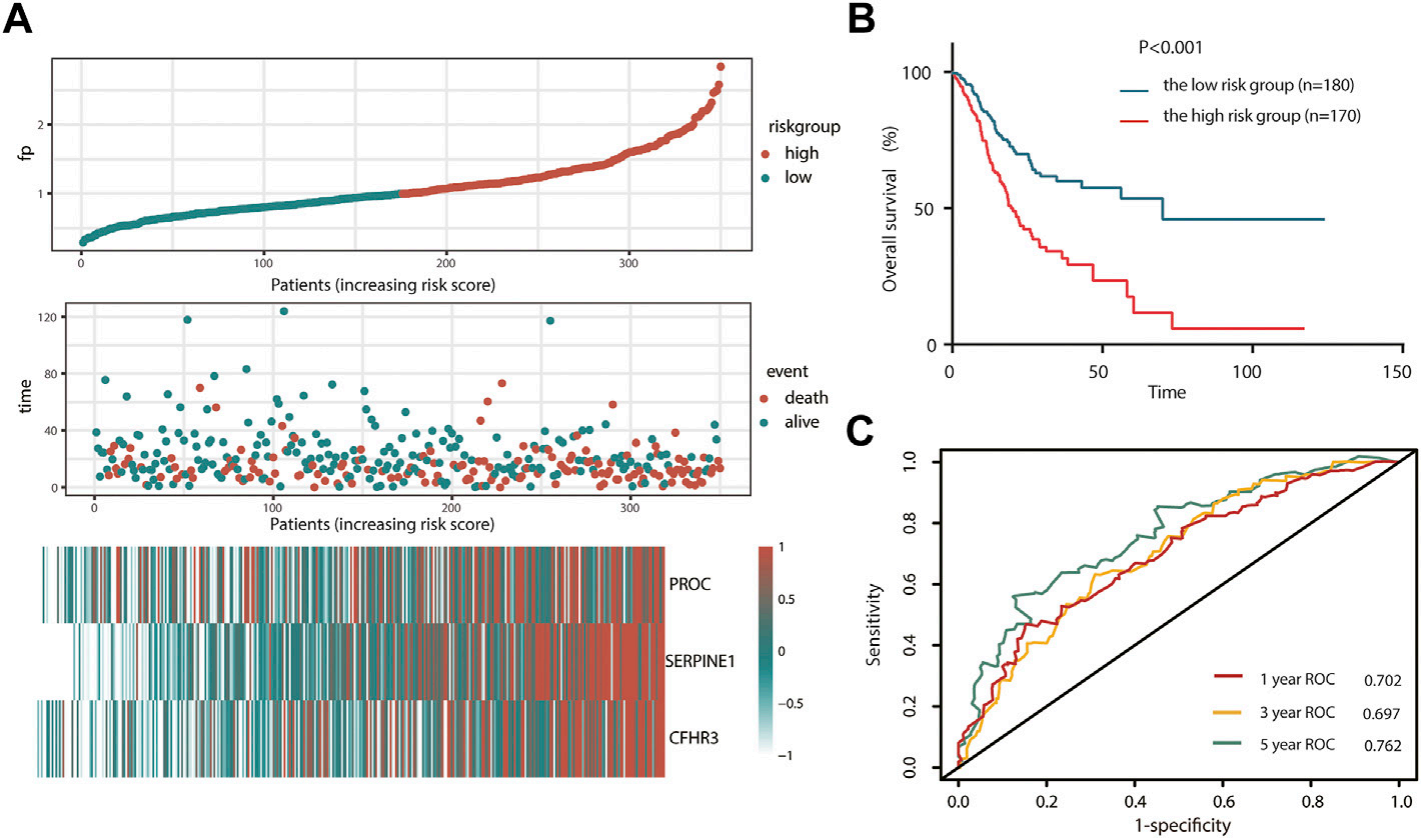
Prognosis analysis of complement system-related three-gene signature in TCGA cohort. **(A)** Detailed risk scores, survival status of TCGA STAD patients, and heatmap of three complement system-related genes in TCGA cohort. **(B)** K–M curves for the overall survival of patients in high- and low-risk groups. **(C)** ROC curves. K–M, Kaplan–Meier; ROC, receiver operating characteristic curve.

Here, we adopted a stepwise multivariate Cox regression analysis to find the best performance efficacy predictive model with the lowest AIC value. Finally, a three-gene-based model, including *SERPINE1*, *PROC*, and *CFHR3*, was successfully developed (Table 2). According to K–M analysis, high *SERPINE1*, *PROC*, and *CFHR3* expression was significantly correlated with poor prognosis (Figures 2E–G). The prognostic risk score model was established with the following formula: risk score = expression level of PROC × 0.092 + expression level of SERPINE1×0.202 + expression level of CFHR3 × 0.127. The results obtained were used to classify patients into low-risk (*n* = 180) and high-risk (*n* = 170) groups based on the optimal cut-off of the risk score, which was calculated by the surv_cutpoint function in the “survminer” package. As shown in Figure 3A, patients in the high-risk group had more occurrences of death and shorter survival times. In addition, the K–M curve indicated that patients in the high-risk group had a significantly worse overall survival than their low-risk counterpart (Figure 3B). Furthermore, the risk heatmap clearly shows that *SERPINE1*, *PROC*, and *CFHR3* were upregulated as the risk score increases (Figure 3A). Time-dependent ROC curves showed that the classifier had a strong predictive ability in TCGA dataset (Figure 3C), the AUC was 0.702 in 1 year, 0.697 in 3 years, and 0.762 in 5 years. Moreover, the risk score model was an independent factor in both univariate and multivariate Cox regression analyses (*p* < 0.001, HR: 2.270, and 95% CI: 1.619–3.183; and *p* < 0.001, HR: 1.999, and 95% CI: 1.384–2.885, respectively) (Table 3).

**TABLE 2:** Results of the stepwise multivariate Cox proportional hazards model.

Gene symbol	Coef	HR	HR 95 L	HR 95H	*p*-value	Regulation
*SERPINE1*	0.202	1.224	1.106	1.355	*p* < 0.001	Up
*PROC*	0.092	1.097	1.011	1.19	*p* = 0.026	Up
*CFHR3*	0.127	1.135	1.043	1.235	*p* = 0.003	Up

**TABLE 3:** Univariate analysis and multivariate analysis of the correlation of risk score with the outcomes among stomach adenocarcinoma patients in two cohorts.

Variable	Univariate Cox analysis			Multivariate Cox analysis		
	Coef	HR(95% CI)	*p*-value	Coef	HR(95% CI)	*p*-value
TCGA STAD (overall survival)						
Age	0.524	1.689 (1.206–2.366)	0.002	0.532	1.704 (1.183–2.454)	0.004
Gender	0.281	1.324 (0.930–1.887)	0.12	0.313	1.368 (0.931–2.011)	0.110
T	0.261	1.299 (1.056–1.599)	0.013	0.067	1.070 (0.799–1.433)	0.648
N	0.281	1.325 (1.142–1.538)	< 0.001	0.147	1.158 (0.925–1.449)	0.198
M	0.718	2.051 (1.157–3.636)	0.014	0.543	1.721 (0.811–3.653)	0.157
Pathologic stage	0.407	1.503 (1.2251.843)	< 0.001	0.188	1.207 (0.809–1.801)	0.355
Risk score	0.819	2.270 (1.619–3.183)	< 0.001	0.692	1.999 (1.384–2.885)	<0.001
GSE84437 (overall survival)						
Age	0.288	1.335 (1.012–1.761)	0.041	0.278	1.320 (0.997–1.748)	0.052
Gender	0.227	1.256 (0.927–1.700)	0.141	0.264	1.303 (0.961–1.766)	0.087
T	0.554	1.740 (1.378–2.198)	< 0.001	0.557	1.747 (1.380–2.211)	<0.001
Risk score	0.793	2.211 (1.222–3.999)	0.008	0.361	1.435 (1.028–2.003)	0.033

### Verification of the prognostic risk score model in the GSE84437 cohort

**FIGURE 4:**
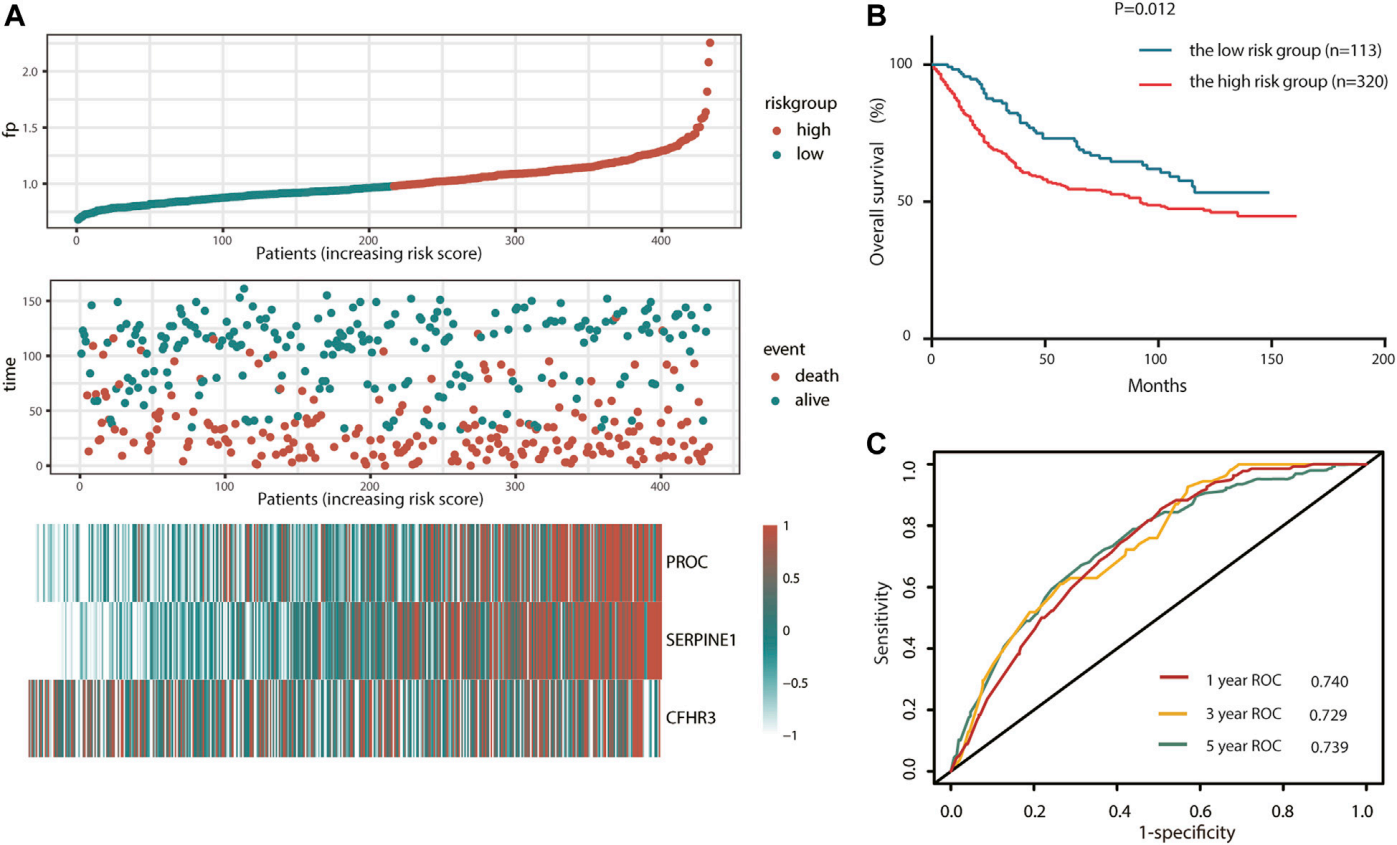
Validation of complement system-related three-gene signature in the GEO cohort. **(A)** Detailed risk scores, survival status of GEO STAD patients, and heatmap of three complement system-related genes in the GEO cohort. **(B)** K–M curves for the overall survival of patients in high- and low-risk groups. **(C)** ROC curves.

To test the robustness of the CSRG signature constructed from TCGA cohort, the risk score of an external validation cohort GSE84437 was calculated using the same formula. Then, the patients from the GSE84437 cohort were also categorized into high-risk (113) or low-risk (320) groups by the optimal cutoff value of the risk score. Consistent with the results obtained from TCGA cohort, patients in the high-risk group had a shorter survival time than those in the low-risk group (Figure 4A). In addition, the high-risk group was significantly associated with poor prognosis (*p* < 0.001, Figure 4B). In addition, the AUC of the signature was 0.740 at 1 year, 0.729 at 3 years, and 0.739 at 5 years (Figure 4C). Also, the risk score model was an independent factor in both the univariate and multivariate Cox analyses (*p* = 0.008, HR: 2.211, and 95% CI: 1.222–3.999; and *p* = 0.033, HR: 1.435, and 95% CI: 1.028–2.003, respectively) (Table 3).

### Landscape of the tumor microenvironment in stomach adenocarcinoma

**FIGURE 5:**
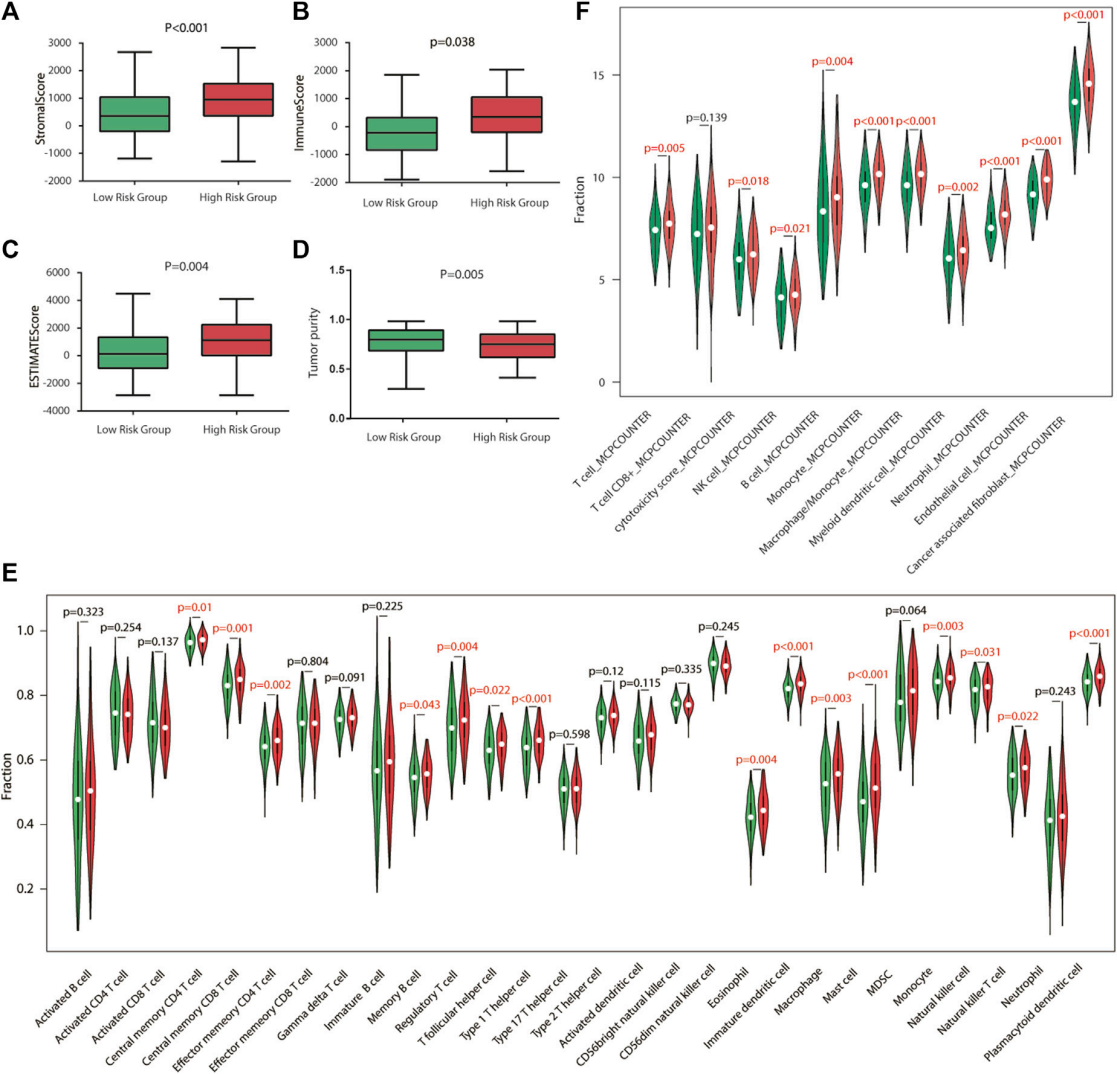
Immune characteristics of TCGA cohort. **(A–D)** Estimation of the proportion of immune-stromal component and tumor purity. Immune score, stromal score, ESTIMATE score (the sum of them), and tumor purity between different risk groups in TCGA cohort. **(E)** Different infiltrating abundances of 28 TIICs estimated by ssGSEA between subgroups. **(F)** Different infiltrating abundances of 11 TIICs estimated by MCP-counter between subgroups. TIICs: tumor-infiltrating immune cells.

**FIGURE 6:**
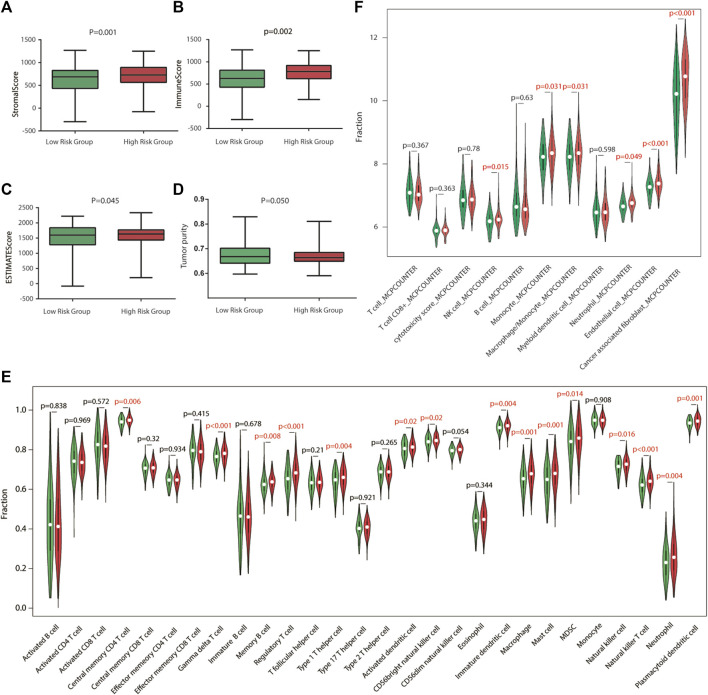
Immune characteristics of the GEO validation cohort. **(A–D)** Estimation of the proportion of immune-stromal component and tumor purity. Immune score, stromal score, ESTIMATE score (the sum of them), and tumor purity between different risk groups in the GEO validation cohort. **(E)** Different infiltrating abundances of 28 TIICs estimated by ssGSEA between subgroups. **(F)** Different infiltrating abundances of 11 TIICs estimated by MCP-counter between subgroups.

To explore the relationship between risk score and TME, we used the ESTIMATE algorithm to determine the four scores of each sample by R software. According to the ESTIMATE algorithm, immune score, stromal score, and ESTIMATE score were significantly higher in high-risk groups, while tumor purity was higher in low-risk groups, indicating that there exist more stromal components in the TME of high-risk groups (Figures 5A–D, 6A–D).

### Landscape of immune infiltration in stomach adenocarcinoma

To analyze the immune status of each sample in TCGA and GSE84437 cohorts, the relative infiltrations of 28 TIICs in the TME were calculated using the SSGSEA algorithm in R. Detailed results are presented in Figures 5,6E. The proportions of immune cells showed differences between groups. Compared with the low-risk group, the high-risk group contained a greater number of central memory CD4 T cells, memory B cells, regulatory T cells, type 1 T helper cells, immature dendritic cells, macrophages, mast cells, natural killer cells, natural killer T cells, and plasmacytoid dendritic cells, implying that their immunological functions associated with the complement system were more active in the high-risk groups. Then, the infiltrating immune cells within TCGA and GSE84437 cohorts were also estimated by MCP-counter methods. For the MCP-counter method, we found that NK cells, monocytes, macrophages, neutrophils, and endothelial cells were elevated in the high-risk groups compared to the low-risk groups (Figures 5,6F).

### Sensitivity of stomach adenocarcinoma patients to chemotherapy agents and checkpoint inhibitors

**FIGURE 7:**
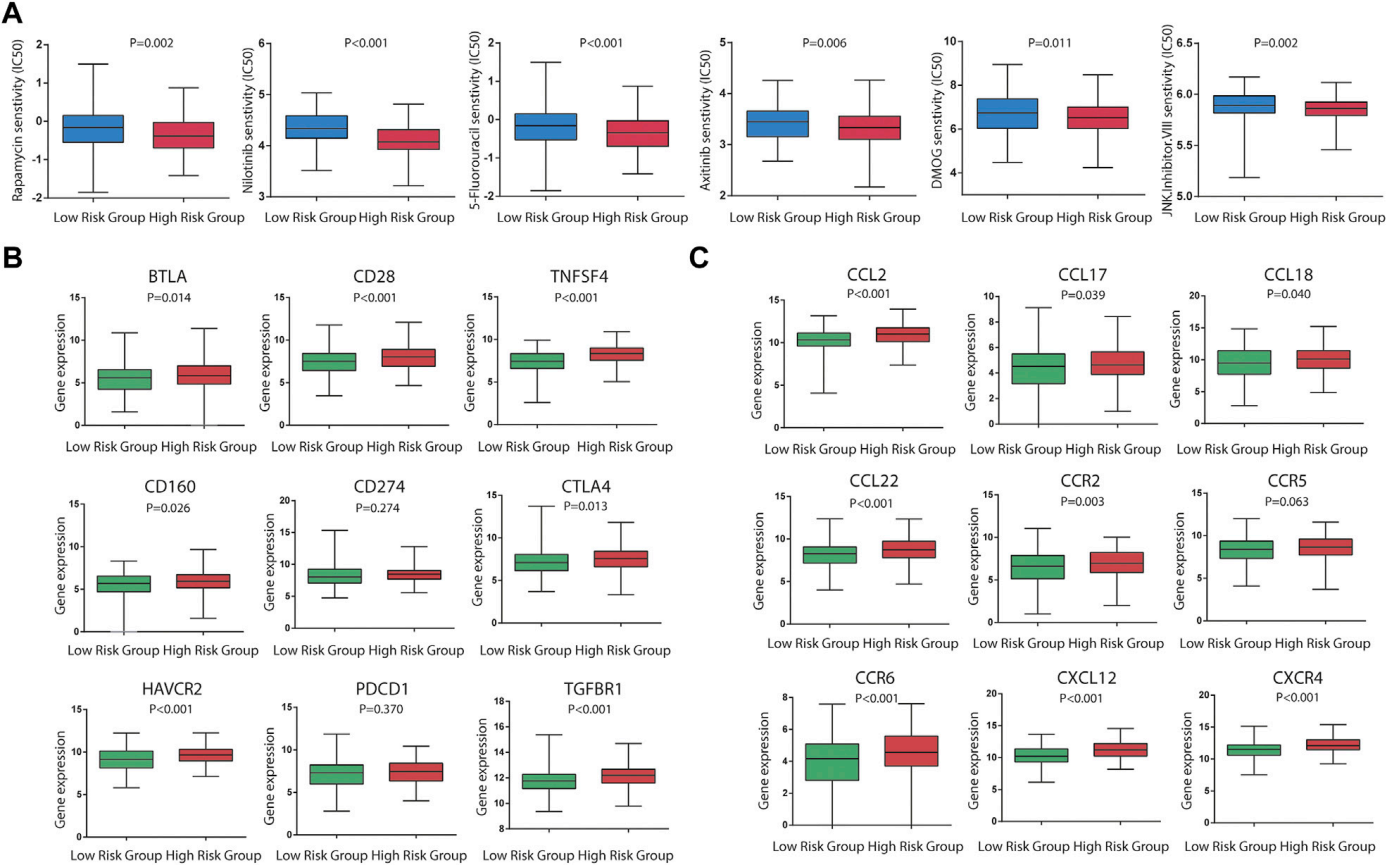
Significance of the CSRG-based signature in chemotherapy and immunotherapy. **(A)** Sensitivity performance of six chemotherapy agents in the high-risk and low-risk subsets (rapamycin, nilotinib, 5-fluorouracil, axitinib, DMOG, and JNK inhibitor VIII). **(B)** Differential expression levels of nine immune checkpoint molecules (CD28, CTLA4, CD274, HAVCR2, BTLA, TNFSF4, CD160, PDCD1, and TGFBR1) between the high-risk and low-risk patients. (C) Differential expression levels of nine immune checkpoint molecules (CD28, CTLA4, CD274, HAVCR2, BTLA,TNFSF4, CD160, PDCD1, and TGFBR1) between the high-risk and low-risk patients. **(C)** Differential expression levels of nine chemokines (CCL2, CCL17, CCL18, CCL22, CCR2, CCR5, CCR6, CXCL12, and CXCR4) between the high-risk and low-risk patients.

**FIGURE 8:**
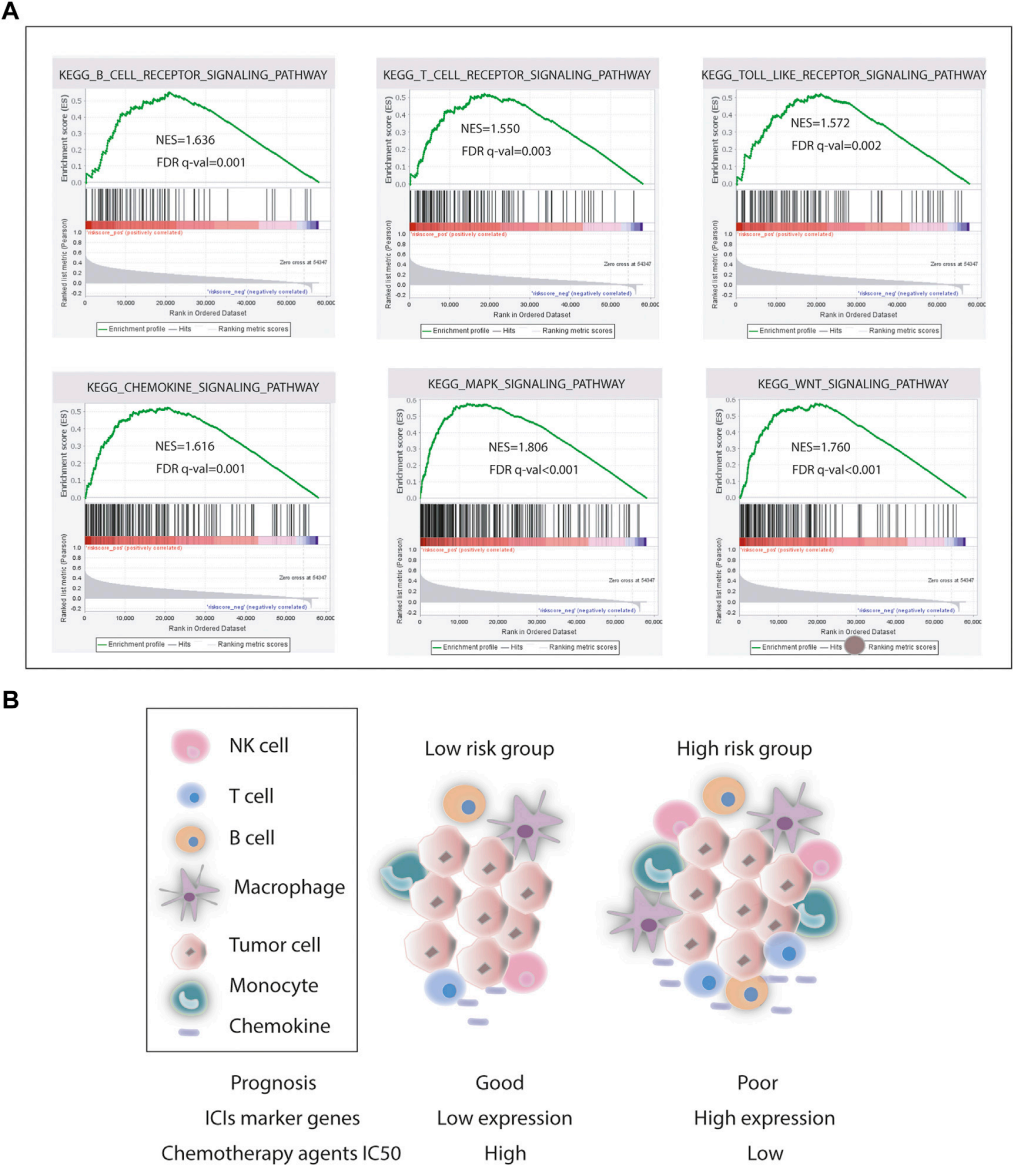
Pathway enrichment analysis. **(A)** Representative pathway enrichment involved in immunity and cancer development. **(B)** Our association analysis of survival, ICI gene expression, chemotherapy agents IC50, and tumor microenvironment in the low- and high-risk groups.

As for chemotherapy sensitivity, the six chemotherapy agents mentioned previously were selected for comparisons of IC50 values between the low- and high-risk groups. STAD high-risk patients showed lower IC50 values for rapamycin, nilotinib, 5-fluorouracil, axitinib, DMOG, and JNK inhibitor VIII (all *p* < 0.05), and these six drugs may be more applicable for patients with a high risk score based on the CSRGs (Figure 7A). Given the significance of immune checkpoint inhibitor-based immunotherapy, the expression levels of nine immune checkpoint molecules (CD28, CTLA4, CD274, HAVCR2, BTLA, TNFSF4, CD160, PDCD1, and TGFBR1) and nine chemokines (CCL2, CCL17, CCL18, CCL22, CCR2, CCR5, CCR6, CXCL12, and CXCR4) that were closely associated with immune cell recruitment between the low- and high-risk groups were compared to evaluate the responses of STAD patients to immunotherapy. Compared with the low-risk group, seven common immune checkpoint molecules had a higher expression in the high-risk subset, but the overexpression of CD274 and PDCD1 was nonsignificant (*p* = 0.274 and *p* = 0.370) (Figure 7B). Also, the expressions of chemokines were all upregulated in the high-risk STAD patients compared with the low-risk patients (all *p* < 0.05) (Figure 7C). These results suggest that the risk model based on CSRGs shows immunotherapy and chemotherapy benefits to STAD patients. In order to elucidate the differences of enriched pathways between the low-risk and high-risk groups, we performed pathway enrichment analysis by the GSEA method. There was obvious immune pathway enrichment in the high-risk group, such as T-cell receptor signaling, B-cell receptor signaling, and cancer promotion pathway such as the MAPK signaling pathway and the Wnt signaling pathway (Figure 8A). Taken together, our observations provided the clues for the association between CSRGs and abundant microenvironment in tumors (Figure 8B).

### Construction and validation of the nomogram

**FIGURE 9:**
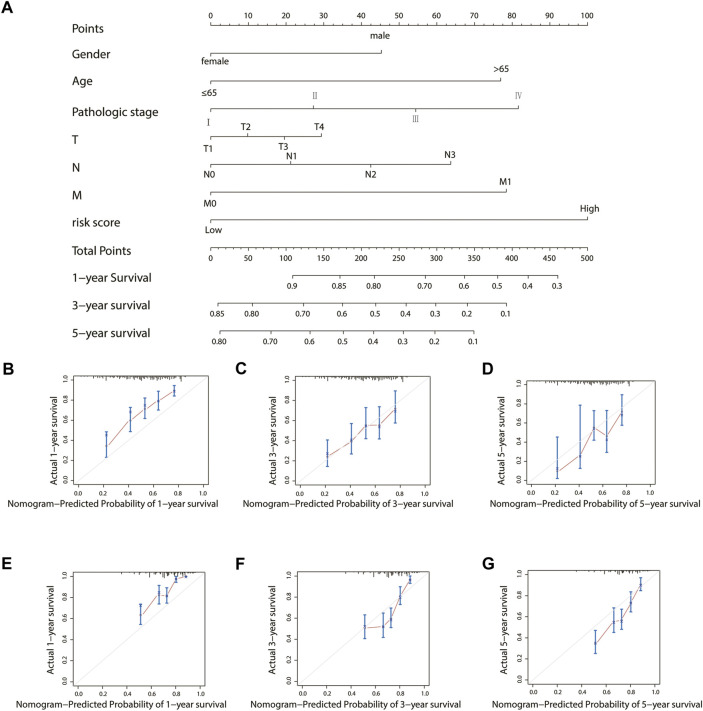
Construction and validation of a nomogram for the overall survival prediction in stomach adenocarcinoma. **(A)** Nomogram of TCGA cohort. **(B–D)** Calibration curves of the nomogram for the estimation of overall survival rates at 1-, 3-, and 5- years in TCGA cohort. **(E–G)** Calibration curves of the nomogram for the estimation of overall survival rates at 1-, 3-, and 5- years in the GEO validation cohort.

To establish a clinically applicable method for predicting the prognosis of stomach adenocarcinoma patients, the risk score and relevant clinical parameters (age, gender, pathologic stage, T, N, and M) were included in the construction of a prognostic nomogram (Figure 9A), and the calculated C-index was 0.700. The nomogram calibration curve was then plotted to compare the predicted overall survival with the observed overall survival. The results showed excellent agreement between the nomogram prediction and actual observation in terms of the 1-, 3-, and 5-year survival rates in both TCGA cohort and the GSE84437 cohort (Figures 9 B–G).

### Exploration of the gene expression *in silico* and in cell lines

**FIGURE 10:**
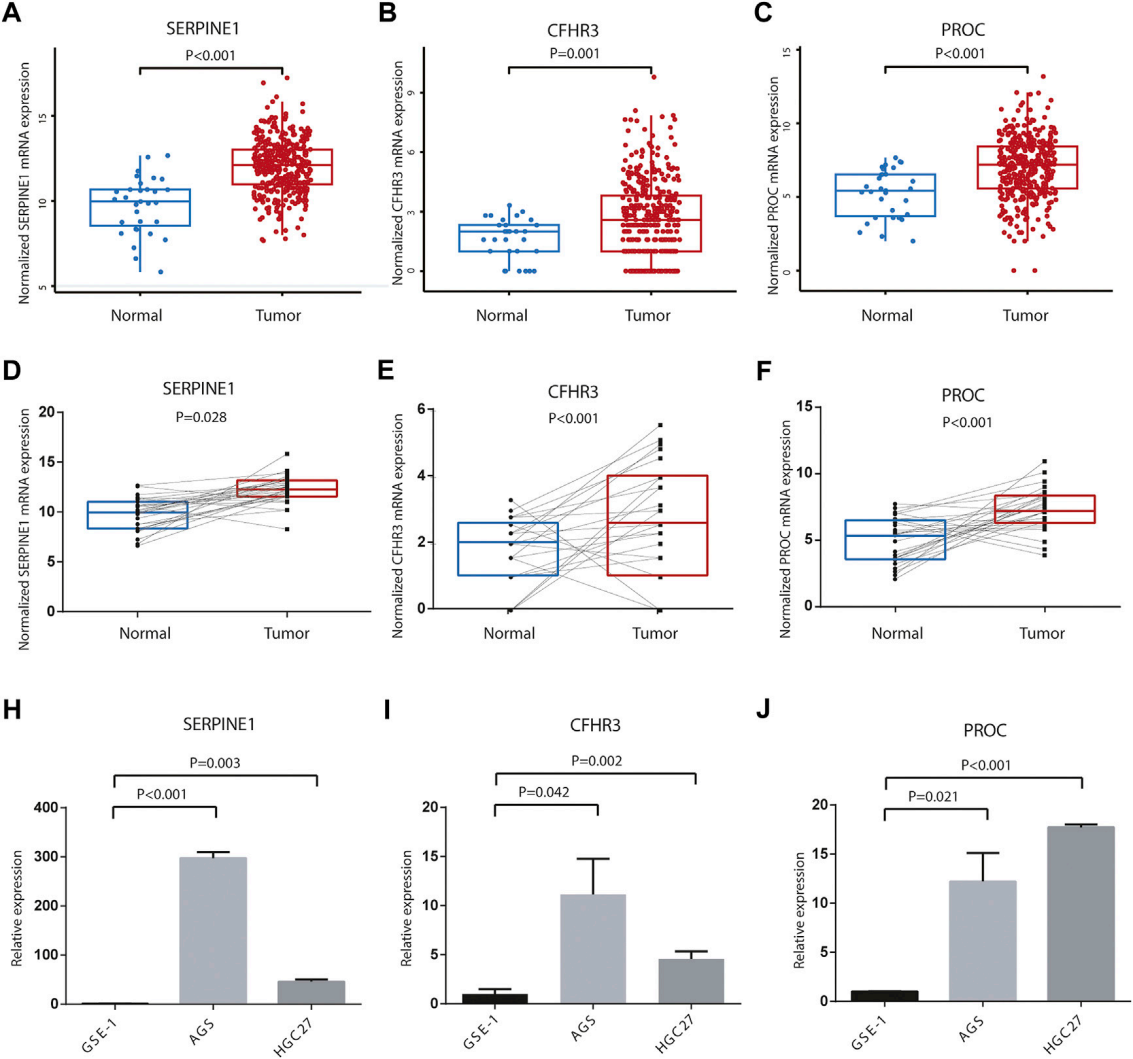
Differences of three-gene expression levels. **(A)** *SERPINE1*, **(B)** *CFHR3*, and **(C)** *PROC* mRNA expression levels were significantly different in the unpaired tumor-adjacent normal TCGA STAD samples. **(D)** SERPINE1, **(E)** CFHR3, and **(F)** PROC mRNA expression levels were significantly different in the paired tumor verse normal TCGA STAD samples. Relative expression levels of **(H)**SERPINE1, **(I)** CFHR3, and **(J)** PROC between AGS, HGC27, and GES-1.

In TCGA database, the three genes’ mRNA expression was significantly higher in tumor tissues than that in adjacent normal tissues, both in unpaired tumor-adjacent normal STAD samples and in the paired tumor-normal STAD samples (Figures 10A–F). Their expression levels were evaluated in AGS, HGC27, and GES-1 by qRT-PCR. Consistently, compared with GES-1, SERPINE1, PROC, and CHFR3 were significantly upregulated in AGS and HGC27 (Figures 10H–J, *p* < 0.05).

## Discussion

Stomach adenocarcinoma is a common cancer and a major cause of cancer-related deaths worldwide (Sung et al., 2021). The extremely poor prognosis of patients with stomach adenocarcinoma greatly promotes the development of an effective treatment measure (Slomski, 2019). Finding solid prognosis predictors will help the stratification of STAD patients and guide precision medical intervention (Zheng et al., 2020).

In this study, we constructed a three-gene CSRG signature that predicted both the survival and immune microenvironment for TCGA STAD patients. After discovering the survival-related CSRGs using univariate and multivariate Cox regression analyses in TCGA cohort, the stepwise multivariate Cox regression analysis was applied, and a three-gene-based signature was generated, which was related to the outcome of TCGA STAD patients. Then, the constructed model was validated in the GEO STAD cohort. ROC curve analyses indicated that the risk score derived from the gene signature could be more efficient in predicting the overall survival in the 1-, 3-, and 5-year survival. Furthermore, using the ESTIMATE, SSGSEA, and MCP-counter methods, we estimated the risk score from complement system-associated genes that were positively associated with most of immune cell infiltration and stroma components in the TME of STAD. It is well known that the TME is typically characterized into three categories (Sung et al., 2021): immune-inflamed: immune activation and abundant immune cell infiltration (Suzuki et al., 2016), immune-excluded: abundant infiltration of immune cells but could not penetrate the tumor parenchyma because of the retention of stroma surrounding cancer nests (Han et al., 2021), and immune desert: associated with immune tolerance and ignorance, and lack of activated and priming T cells (Lanitis et al., 2017). These three patterns had significantly distinct TME cell infiltration characterization. In the present study, the results showed that the high-risk group had a higher abundance of immune cell infiltration and a larger ratio of stroma component but poorer prognosis. According to these results, it was reasonable to speculate that the TME of the high-risk group was in accordance with the immune-excluded subtype. Although a high infiltration of immune cells was present in the TME, these immune cells were unable to function for the recognition and elimination of cancer cells because they were impeded by the abundant stromal element (Zhang et al., 2020). We further assessed the expression levels of these immunosuppressive gene markers and found that CSRGS is related to immune checkpoint molecules (CD28, CTLA4, HAVCR2, BTLA, TNFSF4, CD160, and TGFBR1) and chemokines (CCL2, CCL17, CCL18, CCL22, CCR2, CCR5, CCR6, CXCL12, and CXCR4). The resistance and sensitivity of chemotherapy agents were analyzed to predict the potential of CSRGs to determine the therapeutic effect. These results indicate that the CSRG signature was a potential model to determine which STAD patients are more inclined to respond to ICIs and chemotherapy agents.

Among the three genes in the risk signature, SERPINE1 protein is one important member of the serine proteinase inhibitor E superfamily, and serine protease inhibitors, termed as serpins, are key regulators of numerous biological pathways that initiate inflammation, coagulation, angiogenesis, apoptosis, extracellular matrix composition, and complement activation responses (Richardson et al., 2006; Chen et al., 2021). It has been reported as the key player for poor prognosis and carcinogenesis (Vachher et al., 2020; Wang et al., 2021). Yang et al. (2019) discovered that SERPINE1 was elevated in the gastric cancer tissues, and its upregulation contributes to the proliferation, invasion, and migration of gastric cancer cells, insinuating that SERPINE1 may be considered as a novel biomarker for gastric cancer treatment. Several studies indicated that SERPINE1 promoted tumor angiogenesis and interacted with inflammatory factors, suggesting that SERPINE1 may be related to the TME (Tan et al., 2021; Teng et al., 2021). Complement factor H-related 3 (CFHR3), belonging to the human factor H protein family, is a major regulator of the complement system, which is associated with various immune system diseases, such as age-related macular degeneration (Schafer et al., 2021), macular degeneration (SanGiovanni et al., 2017), IgA nephropathy (Jullien et al., 2018), atypical hemolytic‐uremic syndrome (Pouw et al., 2018), and systemic lupus erythematosus (Zhao et al., 2011). Yang et al. (2021) reported that the expression of CFHR3 was higher in gallbladder carcinoma tissues than that in control tissues, and higher CFHR3 was significantly correlated with poor prognosis, but its role in stomach adenocarcinoma was hardly presented. Protein C (PROC), a vitamin K-dependent glycoprotein, is one of the natural anticoagulants, which plays an important role in the inhibition of blood coagulation (Winther-Larsen et al., 2020). Hartmut Weiler reported that vascular endothelial cells are one key anatomical locale on which the protein C pathway operates to control complement, fibrinolysis, and vascular permeability, and the protein C pathway regulates complement activation by several mechanisms (Weiler, 2010). Protein C deficiency is a heritable thrombophilia caused by numerous different genetic alterations in the *PROC* gene, and protein C deficiency is diagnosed based on the protein C plasma activity and antigen level (Winther-Larsen et al., 2020). However, to the best of our knowledge, no systematic study of PROC in cancer has been reported.

Unlike the previous gene signature in STAD, our study was the first complement system-associated gene signature, which was thought to be highly associated with the immune status in STAD. It can predict which STAD patients are more inclined to respond to ICIs and chemotherapy agents. Our study would present new insights into the association of the complement system with immune status in STAD research.

However, this preliminary study has several limitations. First, our prognostic model was both constructed and validated with the retrospective data from public databases, and it needs to be verified on a large-scale and multicenter clinical cohort. Second, our findings have to be validated by more *in vitro* and *in vivo* experimental studies. Lastly, although we have tested the predictive effectiveness of our model several times, the intrinsic weakness is still inevitable.

## Conclusion

In summary, a robust complement system-based prognostic risk score named CSRGs was constructed and validated in independent cohorts, and in the following analysis, we concluded that CSRGs and the derived model were significantly associated with immune cell infiltration in STAD, providing new insights on the complement system roles in anticancer immunity.

## Data Availability

The datasets presented in this study can be found in online repositories. The names of the repository/repositories and accession number(s) can be found in the article/Supplementary Material.

## Abbreviations

AUC, area under the curve; CAGE, cap analysis of gene expression and deep sequencing; CSRGs, complement system-related genes; GEO, Gene Expression Omnibus; GSEA, gene set enrichment analysis; HR, hazard ratio; MCP-counter, Microenvironment Cell Populations-counter; OS, overall survival; SAGE, serial analysis of gene expression; ssGSEA, single-sample Gene Set Enrichment Analysis; STAD, stomach adenocarcinoma; TCGA, The Cancer Genome Atlas; TME, tumor microenvironment.
