# Supplementary material for: Complement system-related genes in stomach adenocarcinoma: Prognostic signature, immune landscape, and drug resistance
Source: Front Genet. 2022 Sep 8;13:903421. doi: 10.3389/fgene.2022.903421 (PMC9493128; doi:10.3389/fgene.2022.903421)
Supplement: Supplementary file 1 [file DataSheet1.PDF]

Supplementary Table 1. List of 248 complement system-related genes.

Dataset	Name of pathway[a]	Selected genes[b]	Number of genes
Genecards	Complement Pathway	*C3,C5,C6,C7,C9,C2,C8A,C8B,C8G ,CFB,C1QA,C1QB,C1QC,C1R,C1S ,C4A,CFD,MASP1,MASP2,MBL2,C D55,CFP,C4B,CD59*	28
Genecards	Complement and coagulation cascades	*A2M, C3, CD46, CPB2, F3, LMAN1, PROC, SERPING1, BDKRB1, C3AR1, CD55, CR1, F5, MASP1, PROS1, TFPI, C1QA, C4B, CFB, CR2, F7, MASP2, SERPINA1, THBD, C1QC, C6, CFH, F12, F9, PLAT, SERPINC1, C1QB, C5AR1, CFD, F10, F8, MIR6843, SERPINA5, VWF, C2, C9, CLU, F2R, KNG1, PLG, SERPINF2, C1S, C8G, CLTC, F2, KLKB1, PLAUR, SERPINE1, C1R, C7, CFI, F13B, FGB, PLAU, SERPIND1,*	60
Genecards	Creation of C4 and C2 activators	*FCN1, FCN2, MASP1, FCN3, MASP2, C1QA, C1QB, C1QC, C1R, C1S, CRP, IGHG1, IGHG2, IGHG3, IGHG4, IGLC1, IGLC6, MBL2, IGKV3D-11, IGKV5-2, IGKV4-1, CFB, C3, CFD, C4B, COLEC10, COLEC11, C4B_2, C3AR1, C4BPA, C4BPB, C5AR1, CD59, CR1, CD55, CFH, GZMM, C5, C6, C7, C8A, C8B, C8G, C9, CLU, MIR6843, C2, C4A, CFI, CD46, PROS1, VTN, CFHR3, C5AR2,*	54
HGNC	Complement system	*C1R, C1S, C2, C3, C5, C6, C7, C9, CFB, CFD, CFH, CFI, CFP, C1QA, C1QB, C1QBP, C1QC, C1QL1, C1QL2, C1QL3, C1QL4, C1RL, C3AR1, C5AR1, C5AR2, C8A, C8B, C8G, CR2, C3P1, C4A, C4B, CFHR1, CFHR2, CFHR3, CFHR4, CFHR5, C1QBPP1, C1QBPP2, C1QBPP3, C1QL1P1, C4BPA, C4BPB, CR1L, C4B_2, CR1, A1CF, GLYB, AF8T, C1HR, FANCA, FANCB, FANCC, FANCD2, FANCE, FANCF, FANCG, FANCI,*	115

		FANCL, FANCM, K12T, XRCC1, XRCC2, XRCC3, XRCC4, XRCC5, XRCC6, XRCC8, ERCM1, XRCC6P1, XRCC6P2, XRCC6P3, XRCC6P4, XRCC6P5, XRCC4L, RGCC, VSIG4, C4BPAP2, C4BPAP1, CD55, ITGAX, C4BPAP3, ITGB2, VTN, C1QTNF4, CD46P1, ITGAM, CD46, CD59, C1QTNF5, CD93, CLU, ERCC2, ERCC5, ERCC3, ERCC4, ERCC6, RAD23B, ERCC6L, ERCC1, ERCC8, ERCC6L2, UBA1, PALB2, SLX4, XPA, XPC, COG1, COG2, FANCD2P1, FANCD2P2, ATM, BRCA2, POLR3D, BRCA1,	
Gene Set Enrichment Analysis	GOBP_COMPLEMENT_ACTIVATION	A2M, APCS, C1QA, C1QB, C1QBP, C1QC, C1R, C1RL, C1S, C2, C3, C4A, C4B, C4BPA, C4BPB, C5, C6, C7, C8A, C8B, C8G, C9, CD46, CD55, CD59, CD5L, CFB, CFD, CFH, CFHR1, CFHR5, CFI, CFP, CLU, COLEC10, COLEC11, CR1, CR1L, CR2, CRP, FCN1, FCN2, FCN3, IGHA1, IGHA2, IGHD, IGHE, IGHG1, IGHG2, IGHG3, IGHG4, IGHM, IGHV1-18, IGHV1-24, IGHV1-3, IGHV1-45, IGHV1-58, IGHV1-69, IGHV1-69-2, IGHV1-69D, IGHV1OR15-1, IGHV2-26, IGHV2-5, IGHV2-70, IGHV2-70D, IGHV3-11, IGHV3-13, IGHV3-15, IGHV3-16, IGHV3-20, IGHV3-21, IGHV3-23, IGHV3-30, IGHV3-33, IGHV3-35, IGHV3-38, IGHV3-43, IGHV3-48, IGHV3-49, IGHV3-53, IGHV3-64, IGHV3-64D, IGHV3-66, IGHV3-7, IGHV3-72, IGHV3-73, IGHV3-74, IGHV4-28, IGHV4-31, IGHV4-34, IGHV4-39, IGHV4-4, IGHV4-59, IGHV4-61, IGHV5-10-1, IGHV5-51, IGHV6-1, IGHV7-4-1, IGHV7-81, IGKC, IGLC1, IGLC2, IGLC3, IGLC6, IGLC7, IGLL1, IGLL5, IL1B, KRT1, MASP1, MASP2, MBL2, MFAP4, MIR520B, MIR520E, PHB, PHB2, RGCC, SERPING1, SUSD4, TRBC1, TRBC2, TRDC, TREM2, VSIG4,	125
Total			248
[a] Genes were selected based on online datasets and literatures;			

[b] Duplicated genes had been removed<br>Keyword: complement<br>Organism: Homo sapiens

Supplementary Table2. the sequence information of gene primers.

primers for genes	sequence
PROC - FORWARD	CTGCACGCATTACTGCCTAGA
PROC - REVERSE	CCTCCCACAAGGGAACTTCA
CFHR3 - FORWARD	AGTCCGTCAGACCACAGTTAC
CFHR3 - REVERSE	CTGCTGTTGCATATCCTGGTTTA
SERPINE1 - FORWARD	ACCGCAACGTGGTTTTCTCA
SERPINE1 - REVERSE	TTGAATCCCATAGCTGCTTGAAT
